# Supplementary material for: Present-day central African forest is a legacy of the 19th century human history
Source: eLife. 2017 Jan 17;6:e20343. doi: 10.7554/eLife.20343 (PMC5241113; doi:10.7554/eLife.20343)
Supplement: Supplementary file 1. — Botanical family was extracted from the African Plant Database of the Conservatoire et Jardin botaniques de la Ville de Genève and South African National Biodiversity Institute, Pretoria (http://www.ville-ge.ch/musinfo/bd/cjb/africa/recherche.php). Trait information, including regeneration guild sensu Hawthorne (1995) (P = pioneers; NPLD = non-pioneer light-demanders; SB = shade-bearers), leaf phenology (deci = deciduous; ever = evergreen) and wood basic density, was extracted from Fayolle et al. (2014b). Diameter distribution was studied across the SRI using an ordination followed by a clustering (Figure 2). Total number of stems, mean diameter (in cm) and basal area (in m²) are given. The four genera that are monospecific in the SRI and were used for age estimations are highlighted. DOI: http://dx.doi.org/10.7554/eLife.20343.008 [file elife-20343-supp1.docx]

**Supplementary file 1**

**Trait information and characteristics of the diameter distribution for the 176 study genera across the SRI.**

Botanical family was extracted from the African Plant Database of the Conservatoire et Jardin botaniques de la Ville de Genève and South African National Biodiversity Institute, Pretoria (<http://www.ville-ge.ch/musinfo/bd/cjb/africa/recherche.php>). Trait information, including regeneration guild *sensu* Hawthorne (1995) (P = pioneers; NPLD = non-pioneer light-demanders; SB = shade-bearers), leaf phenology (deci = deciduous; ever = evergreen) and wood basic density, was extracted from Fayolle et al. (2014b). Diameter distribution was studied across the SRI using an ordination followed by a clustering (Fig. S1). Total number of stems, mean diameter (in cm) and basal area (in m²) are given. The four genera that are monospecific in the SRI and were used for age estimations are highlighted.

|  |  | **Trait (extracted from Fayolle et al. 2014b)** | | | **Diameter distribution**  **(computed from forest inventory data)** | | | |
| --- | --- | --- | --- | --- | --- | --- | --- | --- |
| **Genus** | **Family** | **Regeneration guild** | **Leaf phenology** | **Wood density (g.cm^-3^)** | **Type of diameter distribution** | **# of stems** | **mean D** | **mean G** |
| *Afzelia* | Fabaceae | NPLD | deci | 0.614 | 1. reverse-J shape | 3479 | 43.50 | 0.231 |
| *Allanblackia* | Clusiaceae | SB | ever | 0.524 | 1. reverse-J shape | 2647 | 36.61 | 0.179 |
| *Alstonia* | Apocynaceae | P | deci | 0.298 | 2. deviation from reverse-J | 19968 | 63.77 | 0.459 |
| *Amphimas* | Fabaceae | NPLD | deci | 0.711 | 2. deviation from reverse-J | 6661 | 58.60 | 0.396 |
| *Angylocalyx* | Fabaceae | SB | ever | 0.612 | 1. reverse-J shape | 28982 | 39.63 | 0.216 |
| *Annona* | Annonaceae |  |  | 0.529 | 1. reverse-J shape | 148 | 42.03 | 0.229 |
| *Anonidium* | Annonaceae | SB | ever | 0.529 | 1. reverse-J shape | 32741 | 40.87 | 0.237 |
| *Anopyxis* | Rhizophoraceae | P | ever | 0.725 | 2. deviation from reverse-J | 6914 | 58.23 | 0.376 |
| *Anthocleista* | Loganiaceae | P | ever | 0.586 | 1. reverse-J shape | 720 | 38.96 | 0.180 |
| *Antiaris* | Moraceae | P | deci | 0.378 | 2. deviation from reverse-J | 2390 | 60.74 | 0.434 |
| *Antidesma* | Euphorbiaceae | SB | ever | 0.640 | 1. reverse-J shape | 350 | 32.86 | 0.148 |
| *Antrocaryon* | Anacardiaceae | P | deci | 0.445 | 2. deviation from reverse-J | 1902 | 56.07 | 0.351 |
| *Aoranthe* | Rubiaceae | P | ever | 0.745 | 1. reverse-J shape | 521 | 34.18 | 0.152 |
| *Autranella* | Sapotaceae | NPLD | deci | 0.782 | 2. deviation from reverse-J | 1691 | 93.46 | 0.914 |
| *Baillonella* | Sapotaceae | NPLD | deci | 0.658 | 2. deviation from reverse-J | 71 | 81.27 | 0.754 |
| *Balanites* | Zygophyllaceae | NPLD | ever | 0.586 | 2. deviation from reverse-J | 603 | 64.71 | 0.489 |
| *Barteria* | Passifloraceae | NPLD | deci | 0.640 | 1. reverse-J shape | 167 | 38.26 | 0.192 |
| *Beilschmiedia* | Lauraceae | SB | ever | 0.586 | 1. reverse-J shape | 11902 | 47.80 | 0.280 |
| *Berlinia* | Fabaceae | SB | ever | 0.614 | 1. reverse-J shape | 3367 | 41.48 | 0.264 |
| *Blighia* | Sapindaceae | SB | ever | 0.632 | 1. reverse-J shape | 15223 | 46.63 | 0.266 |
| *Bobgunnia* | Fabaceae | NPLD | ever | 0.876 | 1. reverse-J shape | 894 | 47.35 | 0.253 |
| *Bombax* | Malvaceae | P | deci | 0.382 | 2. deviation from reverse-J | 1447 | 67.30 | 0.507 |
| *Brenania* | Rubiaceae | P | ever | 0.586 | 1. reverse-J shape | 1342 | 44.30 | 0.240 |
| *Bridelia* | Euphorbiaceae | P |  | 0.640 | 1. reverse-J shape | 821 | 41.35 | 0.196 |
| *Canarium* | Burseraceae | P | deci | 0.418 | 2. deviation from reverse-J | 1973 | 66.60 | 0.493 |
| *Carapa* | Meliaceae | SB | ever | 0.702 | 1. reverse-J shape | 1162 | 36.49 | 0.168 |
| *Cassia* | Fabaceae |  |  | 0.614 | 1. reverse-J shape | 494 | 44.01 | 0.221 |
| *Ceiba* | Malvaceae | P | deci | 0.275 | 2. deviation from reverse-J | 4020 | 92.32 | 0.916 |
| *Celtis* | Ulmaceae | NPLD | deci | 0.602 | 1. reverse-J shape | 109557 | 44.43 | 0.341 |
| *Chionanthus* | Oleaceae |  |  | 0.586 | 2. deviation from reverse-J | 48 | 57.50 | 0.335 |
| *Chlamydocola* | Malvaceae | SB | ever | 0.484 | 1. reverse-J shape | 84 | 41.43 | 0.339 |
| *Christiana* | Malvaceae |  |  | 0.484 | 1. reverse-J shape | 309 | 35.31 | 0.173 |
| *Chrysophyllum* | Sapotaceae | SB | ever | 0.658 | 1. reverse-J shape | 28439 | 47.18 | 0.287 |
| *Citropsis* | Rutaceae | SB | ever | 0.507 | 1. reverse-J shape | 41 | 33.41 | 0.143 |
| *Coelocaryon* | Myristicaceae | SB | ever | 0.446 | 1. reverse-J shape | 10577 | 38.75 | 0.206 |
| *Cola* | Malvaceae | SB | ever | 0.484 | 1. reverse-J shape | 13209 | 38.83 | 0.203 |
| *Copaifera* | Fabaceae | NPLD | ever | 0.614 | 2. deviation from reverse-J | 2421 | 71.51 | 0.548 |
| *Cordia* | Boraginaceae | P | deci | 0.586 | 2. deviation from reverse-J | 1976 | 62.04 | 0.450 |
| *Croton* | Euphorbiaceae | P | deci | 0.640 | 1. reverse-J shape | 6928 | 44.64 | 0.274 |
| *Dacryodes* | Burseraceae | NPLD | ever | 0.541 | 1. reverse-J shape | 3695 | 38.91 | 0.200 |
| *Daniellia* | Fabaceae | SB | deci | 0.614 | 2. deviation from reverse-J | 1081 | 67.23 | 0.535 |
| *Desplatsia* | Malvaceae | SB | ever | 0.484 | 1. reverse-J shape | 4250 | 34.59 | 0.161 |
| *Detarium* | Fabaceae | P | deci | 0.565 | 2. deviation from reverse-J | 2212 | 76.90 | 0.641 |
| *Dialium* | Fabaceae | SB | ever | 0.614 | 1. reverse-J shape | 26618 | 43.70 | 0.271 |
| *Diospyros* | Ebenaceae | SB | ever | 0.658 | 1. reverse-J shape | 26809 | 40.70 | 0.244 |
| *Discoglypremna* | Euphorbiaceae | P | deci | 0.337 | 1. reverse-J shape | 7031 | 37.09 | 0.176 |
| *Dracaena* | Liliaceae | NPLD | ever | 0.586 | 2. deviation from reverse-J | 1853 | 58.92 | 0.427 |
| *Drypetes* | Euphorbiaceae | SB | ever | 0.640 | 1. reverse-J shape | 24888 | 39.27 | 0.214 |
| *Duboscia* | Malvaceae | NPLD | ever | 0.484 | 1. reverse-J shape | 25736 | 49.55 | 0.305 |
| *Elaeis* | Arecaceae | P | ever | 0.586 | 1. reverse-J shape | 365 | 34.11 | 0.351 |
| *Endodesmia* | Clusiaceae |  | ever | 0.635 | 1. reverse-J shape | 153 | 40.52 | 0.189 |
| *Entandrophragma* | Meliaceae | NPLD | deci | 0.494 | 2. deviation from reverse-J | 35442 | 74.67 | 0.643 |
| *Eribroma* | Malvaceae | SB | deci | 0.484 | 1. reverse-J shape | 11965 | 49.08 | 0.289 |
| *Erismadelphus* | Vochysiaceae |  | ever | 0.568 | 1. reverse-J shape | 218 | 48.35 | 0.294 |
| *Erythrina* | Fabaceae | P | deci | 0.612 | 2. deviation from reverse-J | 72 | 53.47 | 0.327 |
| *Erythrophleum* | Fabaceae | P | deci | 0.774 | 2. deviation from reverse-J | 19518 | 67.36 | 0.488 |
| *Fernandoa* | Bignoniaceae | P | deci | 0.586 | 1. reverse-J shape | 1640 | 42.15 | 0.207 |
| *Ficus* | Moraceae | P |  | 0.547 | 2. deviation from reverse-J | 5740 | 70.57 | 0.558 |
| *Fillaeopsis* | Fabaceae | NPLD | deci | 0.488 | 1. reverse-J shape | 365 | 47.18 | 0.304 |
| *Funtumia* | Apocynaceae | SB | ever | 0.586 | 1. reverse-J shape | 21461 | 35.00 | 0.176 |
| *Ganophyllum* | Sapindaceae | SB | ever | 0.507 | 1. reverse-J shape | 9521 | 45.60 | 0.278 |
| *Garcinia* | Clusiaceae | SB | ever | 0.640 | 1. reverse-J shape | 4324 | 31.83 | 0.139 |
| *Gilbertiodendron* | Fabaceae | SB | ever | 0.614 | 1. reverse-J shape | 16776 | 55.54 | 0.672 |
| *Gilletiodendron* | Fabaceae | SB | ever | 0.614 | 1. reverse-J shape | 269 | 43.68 | 0.265 |
| *Gmelina* | Verbenaceae |  |  | 0.586 | 1. reverse-J shape | 60 | 46.17 | 0.226 |
| *Grewia* | Malvaceae | SB | ever | 0.484 | 1. reverse-J shape | 9328 | 37.04 | 0.185 |
| *Guarea* | Meliaceae | SB | ever | 0.511 | 1. reverse-J shape | 16329 | 46.18 | 0.284 |
| *Hallea* | Rubiaceae | NPLD | ever | 0.491 | 1. reverse-J shape | 3774 | 50.79 | 0.376 |
| *Harungana* | Clusiaceae | P | ever | 0.640 | 1. reverse-J shape | 277 | 32.92 | 0.144 |
| *Hexalobus* | Annonaceae | SB | ever | 0.529 | 1. reverse-J shape | 14392 | 45.18 | 0.246 |
| *Holoptelea* | Ulmaceae | P | deci | 0.545 | 2. deviation from reverse-J | 1090 | 57.74 | 0.351 |
| *Homalium* | Salicaceae | SB | ever | 0.640 | 1. reverse-J shape | 9909 | 36.92 | 0.176 |
| *Hylodendron* | Fabaceae | P | ever | 0.721 | 1. reverse-J shape | 2631 | 42.97 | 0.254 |
| *Hymenocardia* | Euphorbiaceae | P | ever | 0.640 | 1. reverse-J shape | 2271 | 35.89 | 0.223 |
| *Irvingia* | Irvingiaceae | SB | ever | 0.640 | 2. deviation from reverse-J | 25634 | 59.73 | 0.438 |
| *Isolona* | Annonaceae | SB | ever | 0.529 | 1. reverse-J shape | 5435 | 35.44 | 0.175 |
| *Julbernardia* | Fabaceae | SB | ever | 0.614 | 1. reverse-J shape | 70 | 36.29 | 0.227 |
| *Keayodendron* | Euphorbiaceae | SB | ever | 0.640 | 1. reverse-J shape | 9517 | 44.72 | 0.253 |
| *Khaya* | Meliaceae | NPLD | deci | 0.538 | 2. deviation from reverse-J | 2460 | 68.17 | 0.520 |
| *Klainedoxa* | Irvingiaceae | P | deci | 0.640 | 2. deviation from reverse-J | 10269 | 67.35 | 0.503 |
| *Lasiodiscus* | Rhamnaceae | SB | ever | 0.547 | 1. reverse-J shape | 87 | 32.53 | 0.119 |
| *Lepisanthes* | Sapindaceae |  |  | 0.507 | 1. reverse-J shape | 49 | 40.41 | 0.179 |
| *Librevillea* | Fabaceae |  |  | 0.614 | 1. reverse-J shape | 81 | 48.15 | 0.320 |
| *Lophira* | Ochnaceae | P | deci | 0.864 | 2. deviation from reverse-J | 8322 | 61.66 | 0.508 |
| *Lovoa* | Meliaceae | NPLD | deci | 0.440 | 2. deviation from reverse-J | 3021 | 64.29 | 0.476 |
| *Macaranga* | Euphorbiaceae | P | ever | 0.640 | 1. reverse-J shape | 30959 | 37.00 | 0.214 |
| *Maesobotrya* | Euphorbiaceae | SB | ever | 0.640 | 1. reverse-J shape | 59 | 32.71 | 0.180 |
| *Mammea* | Clusiaceae | SB | ever | 0.586 | 2. deviation from reverse-J | 3413 | 58.91 | 0.387 |
| *Manilkara* | Sapotaceae | SB | ever | 0.658 | 1. reverse-J shape | 54270 | 48.80 | 0.387 |
| *Mansonia* | Malvaceae | NPLD | deci | 0.550 | 1. reverse-J shape | 8222 | 45.31 | 0.301 |
| *Maranthes* | Chrysobalanaceae | SB | ever | 0.640 | 1. reverse-J shape | 4322 | 50.45 | 0.300 |
| *Mareyopsis* | Euphorbiaceae | SB | ever | 0.640 | 1. reverse-J shape | 78 | 33.59 | 0.125 |
| *Meiocarpidium* | Annonaceae | SB | ever | 0.529 | 1. reverse-J shape | 334 | 31.62 | 0.158 |
| *Milicia* | Moraceae | P | deci | 0.547 | 2. deviation from reverse-J | 3279 | 66.03 | 0.492 |
| *Millettia* | Fabaceae | P | deci | 0.612 | 1. reverse-J shape | 19527 | 47.57 | 0.334 |
| *Mimusops* | Sapotaceae |  |  | 0.658 | 1. reverse-J shape | 48 | 38.33 | 0.183 |
| *Morinda* | Rubiaceae | P | ever | 0.586 | 1. reverse-J shape | 555 | 40.00 | 0.188 |
| *Morus* | Moraceae | NPLD | deci | 0.725 | 2. deviation from reverse-J | 465 | 52.04 | 0.322 |
| *Musanga* | Urticaceae | P | ever | 0.250 | 1. reverse-J shape | 22656 | 43.62 | 0.337 |
| *Myrianthus* | Rubiaceae | NPLD | ever | 0.586 | 1. reverse-J shape | 18105 | 36.04 | 0.218 |
| *Nauclea* | Rubiaceae | P | ever | 0.586 | 1. reverse-J shape | 2978 | 53.30 | 0.326 |
| *Neosloetiopsis* | Moraceae | SB | ever | 0.547 | 1. reverse-J shape | 280 | 35.86 | 0.148 |
| *Newtonia* | Euphorbiaceae |  | ever | 0.640 | 2. deviation from reverse-J | 124 | 50.48 | 0.280 |
| *Oddoniodendron* | Fabaceae | SB | ever | 0.614 | 1. reverse-J shape | 120 | 38.17 | 0.257 |
| *Odyendyea* | Simaroubaceae | NPLD | ever | 0.507 | 1. reverse-J shape | 944 | 44.85 | 0.290 |
| *Olax* | Olacaceae | SB | ever | 0.586 | 1. reverse-J shape | 549 | 38.14 | 0.178 |
| *Oldfieldia* | Euphorbiaceae |  |  | 0.743 | 1. reverse-J shape | 1275 | 51.38 | 0.300 |
| *Omphalocarpum* | Sapotaceae | SB | ever | 0.658 | 1. reverse-J shape | 3067 | 43.14 | 0.226 |
| *Oncoba* | Salicaceae | P | ever | 0.640 | 1. reverse-J shape | 1736 | 32.80 | 0.145 |
| *Ongokea* | Olacaceae | NPLD | ever | 0.715 | 1. reverse-J shape | 10958 | 52.60 | 0.302 |
| *Ormocarpum* | Fabaceae | NPLD |  | 0.612 | 1. reverse-J shape | 39 | 47.95 | 0.254 |
| *Pachyelasma* | Fabaceae | SB | deci | 0.614 | 2. deviation from reverse-J | 1344 | 89.46 | 0.851 |
| *Pancovia* | Sapindaceae | SB | ever | 0.507 | 1. reverse-J shape | 8409 | 31.79 | 0.153 |
| *Panda* | Pandaceae | SB | ever | 0.640 | 1. reverse-J shape | 19626 | 42.50 | 0.237 |
| *Parinari* | Chrysobalanaceae | NPLD | ever | 0.640 | 1. reverse-J shape | 5701 | 50.76 | 0.350 |
| *Parkia* | Fabaceae | NPLD | deci | 0.574 | 1. reverse-J shape | 1094 | 51.95 | 0.312 |
| *Pausinystalia* | Rubiaceae | SB | ever | 0.586 | 1. reverse-J shape | 11342 | 39.58 | 0.222 |
| *Pentaclethra* | Fabaceae | NPLD | ever | 0.793 | 1. reverse-J shape | 23674 | 51.85 | 0.360 |
| *Pericopsis* | Fabaceae | P | deci | 0.612 | 2. deviation from reverse-J | 1343 | 59.81 | 0.442 |
| *Petersianthus* | Lecythidaceae | NPLD | deci | 0.662 | 1. reverse-J shape | 56233 | 49.93 | 0.347 |
| *Picralima* | Apocynaceae | SB | ever | 0.586 | 1. reverse-J shape | 1449 | 36.12 | 0.180 |
| *Piptadeniastrum* | Fabaceae | NPLD | deci | 0.587 | 2. deviation from reverse-J | 10030 | 72.94 | 0.583 |
| *Plagiosiphon* | Fabaceae | SB | ever | 0.614 | 2. deviation from reverse-J | 42 | 58.10 | 0.383 |
| *Plagiostyles* | Euphorbiaceae | NPLD | ever | 0.640 | 1. reverse-J shape | 4623 | 37.93 | 0.301 |
| *Polyalthia* | Annonaceae | SB | ever | 0.529 | 1. reverse-J shape | 65553 | 33.22 | 0.221 |
| *Polyscias* | Araliaceae | P |  | 0.586 | 1. reverse-J shape | 881 | 34.05 | 0.232 |
| *Pouteria* | Sapotaceae | NPLD | ever | 0.658 | 1. reverse-J shape | 3088 | 51.61 | 0.320 |
| *Prioria* | Fabaceae | NPLD | deci | 0.614 | 2. deviation from reverse-J | 10446 | 64.78 | 0.474 |
| *Pseudospondias* | Anacardiaceae | P | ever | 0.507 | 1. reverse-J shape | 2499 | 42.46 | 0.255 |
| *Psydrax* | Rubiaceae | P | deci | 0.586 | 1. reverse-J shape | 1060 | 40.25 | 0.195 |
| *Pteleopsis* | Combretaceae | P | deci | 0.586 | 2. deviation from reverse-J | 7029 | 59.66 | 0.386 |
| *Pterocarpus* | Fabaceae | NPLD | deci | 0.612 | 1. reverse-J shape | 20563 | 50.59 | 0.295 |
| *Pterygota* | Malvaceae | NPLD | deci | 0.484 | 1. reverse-J shape | 6778 | 47.65 | 0.282 |
| *Pycnanthus* | Myristicaceae | NPLD | ever | 0.414 | 1. reverse-J shape | 19046 | 51.09 | 0.318 |
| *Rauvolfia* | Apocynaceae | P | ever | 0.586 | 1. reverse-J shape | 1075 | 44.20 | 0.232 |
| *Ricinodendron* | Euphorbiaceae | P | deci | 0.216 | 2. deviation from reverse-J | 14703 | 63.01 | 0.470 |
| *Rinorea* | Violaceae | SB | ever | 0.640 | 1. reverse-J shape | 982 | 33.32 | 0.168 |
| *Rothmannia* | Rubiaceae | SB | ever | 0.586 | 1. reverse-J shape | 631 | 43.28 | 0.246 |
| *Santiria* | Burseraceae | SB | ever | 0.521 | 1. reverse-J shape | 12042 | 39.08 | 0.200 |
| *Schrebera* | Oleaceae |  |  | 0.586 | 1. reverse-J shape | 674 | 49.08 | 0.272 |
| *Scorodophloeus* | Fabaceae | SB | ever | 0.614 | 1. reverse-J shape | 3121 | 38.56 | 0.291 |
| *Scottellia* | Salicaceae | SB | ever | 0.552 | 1. reverse-J shape | 7311 | 40.33 | 0.208 |
| *Shirakiopsis* | Euphorbiaceae | NPLD | ever | 0.640 | 1. reverse-J shape | 1791 | 44.15 | 0.224 |
| *Sorindeia* | Anacardiaceae | SB | ever | 0.507 | 1. reverse-J shape | 741 | 34.78 | 0.147 |
| *Spathodea* | Bignoniaceae | P | deci | 0.586 | 1. reverse-J shape | 69 | 43.33 | 0.215 |
| *Spondianthus* | Phyllanthaceae | SB | ever | 0.640 | 1. reverse-J shape | 31 | 42.26 | 0.222 |
| *Stachyothyrsus* | Fabaceae |  |  | 0.614 | 1. reverse-J shape | 41 | 48.78 | 0.297 |
| *Staudtia* | Myristicaceae | SB | ever | 0.744 | 1. reverse-J shape | 32314 | 41.81 | 0.242 |
| *Stemonocoleus* | Fabaceae | NPLD |  | 0.548 | 2. deviation from reverse-J | 181 | 66.08 | 0.557 |
| *Sterculia* | Malvaceae |  | deci | 0.484 | 1. reverse-J shape | 12098 | 37.28 | 0.215 |
| *Stereospermum* | Bignoniaceae | P | deci | 0.586 | 1. reverse-J shape | 538 | 40.69 | 0.194 |
| *Strephonema* | Combretaceae | SB | ever | 0.586 | 2. deviation from reverse-J | 67 | 59.10 | 0.373 |
| *Strombosia* | Olacaceae | SB | ever | 0.586 | 1. reverse-J shape | 62974 | 39.10 | 0.244 |
| *Strombosiopsis* | Olacaceae | SB | ever | 0.586 | 1. reverse-J shape | 24582 | 40.30 | 0.221 |
| *Symphonia* | Clusiaceae | SB | ever | 0.541 | 1. reverse-J shape | 298 | 42.35 | 0.203 |
| *Synsepalum* | Sapotaceae | SB | ever | 0.658 | 1. reverse-J shape | 9921 | 34.99 | 0.171 |
| *Syzygium* | Myrtaceae | SB | ever | 0.586 | 1. reverse-J shape | 2427 | 45.98 | 0.256 |
| *Tabernaemontana* | Apocynaceae |  | ever | 0.586 | 1. reverse-J shape | 700 | 33.81 | 0.156 |
| *Terminalia* | Combretaceae | P | deci | 0.586 | 2. deviation from reverse-J | 34111 | 63.60 | 0.494 |
| *Tessmannia* | Fabaceae | SB | ever | 0.614 | 1. reverse-J shape | 9407 | 52.44 | 0.316 |
| *Tetraberlinia* | Fabaceae | SB | ever | 0.614 | 1. reverse-J shape | 77 | 41.17 | 0.313 |
| *Tetrapleura* | Fabaceae | P | deci | 0.519 | 1. reverse-J shape | 4838 | 42.73 | 0.215 |
| *Treculia* | Moraceae | SB | ever | 0.547 | 1. reverse-J shape | 2836 | 43.63 | 0.223 |
| *Trichilia* | Meliaceae | SB | ever | 0.511 | 1. reverse-J shape | 21051 | 36.46 | 0.188 |
| *Trichoscypha* | Anacardiaceae | NPLD | ever | 0.507 | 1. reverse-J shape | 1063 | 42.33 | 0.225 |
| *Tridesmostemon* | Sapotaceae |  | ever | 0.658 | 1. reverse-J shape | 3471 | 48.35 | 0.289 |
| *Trilepisium* | Moraceae | SB | deci | 0.547 | 1. reverse-J shape | 10582 | 39.59 | 0.236 |
| *Triplochiton* | Malvaceae | P | deci | 0.327 | 2. deviation from reverse-J | 13019 | 87.43 | 0.907 |
| *Uapaca* | Euphorbiaceae | P | ever | 0.640 | 1. reverse-J shape | 9820 | 42.33 | 0.299 |
| *Usteria* | Loganiaceae |  |  | 0.586 | 1. reverse-J shape | 78 | 43.59 | 0.214 |
| *Uvariastrum* | Annonaceae | SB | ever | 0.529 | 1. reverse-J shape | 76 | 34.21 | 0.131 |
| *Vangueriopsis* | Rubiaceae |  |  | 0.586 | 1. reverse-J shape | 41 | 40.00 | 0.261 |
| *Vepris* | Rutaceae | SB | ever | 0.507 | 1. reverse-J shape | 474 | 38.80 | 0.191 |
| *Vitex* | Verbenaceae |  | ever | 0.586 | 1. reverse-J shape | 6737 | 35.64 | 0.164 |
| *Xylopia* | Annonaceae |  | ever | 0.529 | 1. reverse-J shape | 29500 | 38.53 | 0.204 |
| *Zanthoxylum* | Rutaceae | P | ever | 0.507 | 1. reverse-J shape | 7162 | 45.85 | 0.256 |
