# Supplementary material for: Present-day central African forest is a legacy of the 19th century human history
Source: eLife. 2017 Jan 17;6:e20343. doi: 10.7554/eLife.20343 (PMC5241113; doi:10.7554/eLife.20343)
Supplement: Supplementary file 2. — The mode of the diameter distribution across the SRI, information on growth data, including number of trees (n), the diameter (dbh) range and the mean and standard error of the annual diameter increment (SE), and age estimations of the mode based on the mean annual diameter increment (MAId) calculated for 982 monitored trees in the SRI are provided for the four study species (see Figure 2—figure supplement 2). For all study species, eight growth models (i.e., Canham, Gompertz, Verhulst, Power, Power mult, Lognormal, Linear and Mean) were fitted to the data, and age estimations were obtained with numerical solutions to ordinary differential equations (ODE) (see Figure 2—figure supplement 2). The most reliable age estimations according to age data from published tree-ring studies are highlighted. DOI: http://dx.doi.org/10.7554/eLife.20343.009 [file elife-20343-supp2.docx]

**Supplementary file 2**

**Age estimations of the trees at the mode of the diameter distribution for the four genera that are monospecific in the SRI.**

The mode of the diameter distribution across the SRI, information on growth data, including number of trees (n), the diameter (dbh) range and the mean and standard error of the annual diameter increment (SE), and age estimations of the mode based on the mean annual diameter increment (MAI_d_) calculated for 982 monitored trees in the SRI are provided for the four study species. For all study species, eight growth models (i.e., Canham, Gompertz, Verhulst, Power, Power mult, Lognormal, Linear and Mean) were fitted to the data, and age estimations were obtained with numerical solutions to ordinary differential equations (ODE). The most reliable age estimations according to age data from published tree-ring studies are highlighted.

|  |  | **Study species** | | | |
| --- | --- | --- | --- | --- | --- |
|  |  | *E. suaveolens* | *P. elata* | *T. superba* | *T. scleroxylon* |
| **Mode of the distribution** | | | | | |
|  | **Weibull** | 72.0 | 65.3 | 69.5 | 90.3 |
| **Growth data** | | | | | |
|  | **n** | 367 | 199 | 152 | 265 |
|  | **Sites (Fig. 1 Main Text)** | 1, 2, 3, 4, 5, 6, 7 | 3, 4 | 1, 3, 5, 6, 7 | 1, 3, 5, 6 |
|  | **dbh range (cm)** | 12.2 - 151.6 | 14.7 - 104.7 | 10.6 – 80.7 | 10.7 – 149.4 |
|  | **MAI_d_ (mean ± SE)** | 0.44 ± 0.033 | 0.45 ± 0.026 | 0.53 ± 0.112 | 0.58 ± 0.061 |
| **Age estimations (yrs) based on** | | | | | |
|  | **Mean growth (± SE)** | | | | |
| **ODE numerical solving** | **Canham** | **-** | 236 | 177 | 193 |
|  | **Gompertz** | 189 | 223 | 168 | 254 |
|  | **Logistic (Verhulst)** | 444 | 625 | 322 | 866 |
|  | **Power** | - | 48 | - | 76 |
|  | **Power modified exponent** | 129 | 148 | 131 | 170 |
|  | **Power modified multiplier** | 274 | 176 | 129 | 193 |
|  | **Lognormal** | - | 384 | 212 | 223 |
|  | **Linear** | 133 | 152 | 135 | 186 |
|  | **Mean** | 105 | 166 | 86 | 316 |
