# Supplementary material for: Present-day central African forest is a legacy of the 19th century human history
Source: eLife. 2017 Jan 17;6:e20343. doi: 10.7554/eLife.20343 (PMC5241113; doi:10.7554/eLife.20343)
Supplement: Supplementary file 3. — Mean ages and corresponding estimated dates are shown in Figure 2 (Main Text). C = Cameroon; RC = Republic of the Congo; CAR = Central African Republic; n = number of stem discs. DOI: http://dx.doi.org/10.7554/eLife.20343.010 [file elife-20343-supp3.docx]

**Supplementary file 3**

**Age data for the four study species based on published tree-ring data.**

Mean ages and corresponding estimated dates are shown in Fig. 2 (Main Text). C = Cameroon; RC = Republic of the Congo; CAR = Central African Republic; n = number of stem discs.

| **Species** | **Location** | **Date of collection** | **N discs** | **Mean ring width (cm)**  **(cm.yr^-1^)** | **Mean age (yr)** | **Estimated date (AD)** | **References** |
| --- | --- | --- | --- | --- | --- | --- | --- |
| *P. elata* | Kisangani (DRC) | 2008 | 24 | 0.298 ± 0.54 | 137 | 1871 | De Ridder et al., 2014 |
| *T. superba* | Luki & Tschela (DRC) | 2008 | 12 | 0.719 ± 0.267 | 47 | 1961 | De Ridder et al., 2013a; 2013b |
| *T. superba* | Scio (IC) | 2008 | 29 | 0.719 ± 0.267 | 55 | 1954 | De Ridder et al., 2013a; 2013b |
| *T. scleroxylon* | Biakoa (C) | 1993 | 18 | 0.620 ± 0.28 | 124 | 1869 | Worbes al., 2003 |
