# Supplementary material for: Present-day central African forest is a legacy of the 19th century human history
Source: eLife. 2017 Jan 17;6:e20343. doi: 10.7554/eLife.20343 (PMC5241113; doi:10.7554/eLife.20343)
Supplement: Supplementary file 4. — Growth model functions used to analyze the variation in tree growth (MAId, in cm.yr−1) with tree size (DBH, in cm) are detailed below. For the biological interpretation of parameters, Max is the maximum growth or growth optimum (in cm.yr−1), Dopt is the diameter at growth optimum (in cm), and Dmax is the maximum diameter (in cm). Linear and mean models were additionally fitted to the data. For each model fitted to the growth and diameter data of each species, the Bayesian Information Criterion (BIC) is provided. The models with the best performance for each species are highlighted. DOI: http://dx.doi.org/10.7554/eLife.20343.011 [file elife-20343-supp4.docx]

**Supplementary file 4**

**Relative performance of commonly used growth models for the four genera that are monospecific in the SRI.**

Growth model functions used to analyze the variation in tree growth (MAI_d_, in cm.yr^-1^) with tree size (DBH, in cm) are detailed below. For the biological interpretation of parameters, Max is the maximum growth or growth optimum (in cm.yr^-1^), D_opt_ is the diameter at growth optimum (in cm), and D_max_ is the maximum diameter (in cm). Linear and mean models were additionally fitted to the data. For each model fitted to the growth and diameter data of each species, the Bayesian Information Criterion (BIC) is provided. The models with the best performance for each species are highlighted.

| Model | Equation and parameter | | BIC values | | | |
| --- | --- | --- | --- | --- | --- | --- |
|  |  |  | ***E. suaveolens*** | ***P. elata*** | ***T. superba*** | ***T. scleroxylon*** |
| Canham | ${MAI}_{d}=a\times\exp\left[ -\frac{1}{2}\times\left( log\left( \frac{\mathrm{DBH}}{b/c} \right) \right)^{2} \right]$ | a = Max  b = D_opt_  c | 196.6 | -96.8 | 256.1 | 372.1 |
| Gompertz | ${MAI}_{d}=a \times DBH \times log(\frac{b}{DBH})$ | a  b = D_max_ | 219.5 | -98.8 | 277.4 | 397.6 |
| Logistic (Verhulst) | ${MAI}_{d}=a\times DBH \times\left( 1-\frac{DBH}{b} \right)$ | a  b = D_max_ | 246.1 | -90.2 | 289.4 | 398.7 |
| Power | ${MAI}_{d}=a\times{DBH}^{b}$ | a  b | 235.5 | -97.8 | 263.3 | 397.5 |
| Power modified multiplier | ${MAI}_{d}=a\times exp\left( -c\times DBH \right){\times DBH}^{b}$ | a  b  c | 198.2 | -96.3 | 257.8 | 372.1 |
| Lognormal | ${MAI}_{d}=a\times exp\left[ {-\left( b\times\log\left( \frac{c}{DBH} \right) \right)}^{2} \right]$ | a = Max  b  c = D_opt_ | 210.1 | -95.8 | 266.0 | 403.1 |
| Linear | ${MAI}_{d}=a+b\times DBH$ | a  b | 223.5 | -96.5 | 265.0 | 398.1 |
| Mean | ${MAI}_{d}=a$ | a | 241.2 | -99.1 | 267.5 | 411.4 |
