# Supplementary material for: Present-day central African forest is a legacy of the 19th century human history
Source: eLife. 2017 Jan 17;6:e20343. doi: 10.7554/eLife.20343 (PMC5241113; doi:10.7554/eLife.20343)
Supplement: Supplementary file 5. — C = Cameroon; RC = Republic of the Congo; CAR = Central African Republic; W Africa = West Africa. Numbers refer to the map (Figure 1 Main Text). References are indicated. DOI: http://dx.doi.org/10.7554/eLife.20343.012 [file elife-20343-supp5.docx]

**Supplementary file 5**

**Data documenting paleoenvironmental changes (Figs. 1 and 3, Main Text) during the last 1,000 years in the SRI.**

C = Cameroon; RC = Republic of the Congo; CAR = Central African Republic; W Africa = West Africa. Numbers refer to the map (Fig. 1 Main Text). References are indicated.

| **Country** | **Site** | **Fig. 1** | **Latitude** | **Longitude** | **Data type** | **References** |
| --- | --- | --- | --- | --- | --- | --- |
| C | Belabo (upper Sanaga catchment) | 1 | 4.56390 | 13.17200 | Grain size, chemical elements | Sangen et al., 2011 |
| C | Benana C22 (Nyong valley) | 2 | 4.00000 | 13.00000 | Grain size, chemical elements | Runge et al., 2014 |
| C | Mankako (upper Boumba catchment) | 3 | 3.18000 | 14.04000 | Grain size, chemical elements | Sangen et al., 2011 |
| C | Ouesso region (Boumba catchment) | 4 | 3.24000 | 14.32000 | Grain size, chemical elements | Sangen et al., 2011 |
| C | Lobéké Reserve | 5 | 2.17300 | 15.42590 | Phytoliths, δ^13^C | Runge and Fimbel, 2001 |
| C | Moukounounou (upper Ngoko catchment) | 6 | 1.56390 | 15.20090 | Grain size, chemical elements, δ^13^C | Sangen, 2012; Sangen et al., 2011 |
| C | Mokounounou N01 (upper Ngoko catchment) | 6 | 1.56390 | 15.20090 | Grain size, chemical elements, δ^13^C | Sangen, 2012; Sangen et al., 2011 |
| C | Mokounounou N02 (upper Ngoko catchment) | 6 | 1.56390 | 15.20090 | Grain size, chemical elements, δ^13^C | Sangen, 2012; Sangen et al., 2011 |
| C | Mokounounou N04 (upper Ngoko catchment) | 6 | 1.56390 | 15.20090 | Grain size, chemical elements, δ^13^C | Sangen, 2012; Sangen et al., 2011 |
| C | Mokounounou N06 (upper Ngoko catchment) | 6 | 1.56390 | 15.20090 | Grain size, chemical elements, δ^13^C | Sangen, 2012; Sangen et al., 2011 |
| RC | Mopo Bai | 7 | 2.23300 | 16.26200 | Atmospheric dust signal, pollen, μ-charcoal | Brnčić et al., 2009 |
| RC | Mopo Bai Site 1 | 7 | 2.24370 | 16.26190 | Charcoal | Tovar et al., 2014 |
| RC | Mopo Bai Site 8 | 7 | 2.24120 | 16.26130 | Charcoal | Tovar et al., 2014 |
| RC | Mopo Bai Site 9 | 7 | 2.23250 | 16.26240 | Charcoal | Tovar et al., 2014 |
| RC | Mopo Bridge Site 10 | 8 | 2.22720 | 16.28550 | Charcoal | Tovar et al., 2014 |
| RC | Mopo Bridge Site 13 | 8 | 2.21410 | 16.25240 | Charcoal | Tovar et al., 2014 |
| CAR | Mbeli River Site 5 | 9 | 2.28020 | 16.43290 | Charcoal | Tovar et al., 2014 |
| CAR | Mbeli River Site 6 | 9 | 2.27880 | 16.45190 | Charcoal | Tovar et al., 2014 |
| CAR | Mbeli River Site 7 | 9 | 2.27800 | 16.45520 | Charcoal | Harris, 2002 |
| RC | Goualougo Lake | 10 | 2.16400 | 16.50900 | Atmospheric dust signal, pollen, μ-charcoal | Brnčić et al., 2007 |
| CAR | Goualougo Site 14 | 10 | 2.18430 | 16.52040 | Charcoal | Tovar et al., 2014 |
| CAR | Goualougo Site 15 | 10 | 2.16370 | 16.50960 | Charcoal | Tovar et al., 2014 |
| CAR | Goualougo Site 16 | 10 | 2.20280 | 16.50360 | Charcoal | Tovar et al., 2014 |
| RC | Pokola Site 17 | 11 | 1.27080 | 16.79700 | Charcoal | Tovar et al., 2014 |
| RC | Lake Télé | 12 | 1.20000 | 17.10000 | Pollen | Laraque et al, 1998 |
| CAR | Mbaéré valley | 13 | 3.72500 | 17.00000 | Grain size, chemical elements | Neumer et al., 2008 |
| CAR | Sadika alluvial fan | 13 | 3.72500 | 17.00000 | Grain size, chemical elements | Neumer et al., 2008 |
| CAR | Bagbaya (core FC400) | 14 | 3.50000 | 17.28000 | Pollen, charcoal | Lupo et al., 2015 |
| CAR | Ngotto Forest | 15 | 4.00000 | 17.30000 | Remote sensing | Runge, 2008 |
| CAR | Lake Gbali | 16 | 4.81861 | 18.26278 | Charcoal | Aleman et al., 2013 |
| CAR | Oubangui catchment | 17 | 4.62000 | 18.59000 | Flood & erosion data 1911-1999 | Runge and Nguimalet, 2005 |
| CAR | Lake Doukoulou | 18 | 4.25278 | 18.42361 | Charcoal | Aleman et al., 2013 |
| CAR | Lake Nguengué | 19 | 3.76111 | 18.12194 | Charcoal | Aleman et al., 2013 |
| W Africa | Marine core ODP 658C | **Fig. 3** | 20.4500 | -18.35000 | SSTs (foraminifera) | DeMenocal et al., 2000 |
