# Supplementary material for: Present-day central African forest is a legacy of the 19th century human history
Source: eLife. 2017 Jan 17;6:e20343. doi: 10.7554/eLife.20343 (PMC5241113; doi:10.7554/eLife.20343)
Supplement: Supplementary file 6. — C = Cameroon; RC = Republic of the Congo; CAR = Central African Republic; AA = AMS Laboratory, University of Arizona (USA); Beta = Beta Analytic (USA); Erl = Erlangen AMS Facility (Germany); Gif = Gif-sur-Yvette (France); KI = Kiel (Germany); KIA = Kiel AMS (Germany); Ly = University of Lyon (France); OBDY = ORSTOM Bondy (France); Poz = Poznań Radiocarbon Laboratory (Poland). Numbers refer to the map (Figure 1 Main Text). Dated material, identified species, lab codes, raw and calibrated dates Before Present (BP) and Anno Domini (AD), source references, and pottery (i.e. potsherds), smelting (i.e. iron slags and identified tuyères), salt (Richard Oslisly pers. obs.) and charred oil palm endocarps evidence are indicated. We assumed that charred oil palm endocarps found in combination with potsherds were consumed during the occupation of the sites (Morin-Rivat et al., 2016). DOI: http://dx.doi.org/10.7554/eLife.20343.013 [file elife-20343-supp6.docx]

**Supplementary file 6**

**Synthesis of the 63 AMS radiocarbon and the two OSL dates documenting human activities (Fig. 3 Main Text) during the last 1,000 years in the SRI.**

C = Cameroon; RC = Republic of the Congo; CAR = Central African Republic; AA = AMS Laboratory, University of Arizona (USA); Beta = Beta Analytic (USA); Erl = Erlangen AMS Facility (Germany); Gif = Gif-sur-Yvette (France); KI = Kiel (Germany); KIA = Kiel AMS (Germany); Ly = University of Lyon (France); OBDY = ORSTOM Bondy (France); Poz = Poznań Radiocarbon Laboratory (Poland). Numbers refer to the map (Fig. 1 Main Text). Dated material, identified species, lab codes, raw and calibrated dates Before Present (BP) and Anno Domini (AD), source references, and pottery (i.e. potsherds), smelting (i.e. iron slags and identified *tuyères*), salt (Richard Oslisly pers. obs.) and charred oil palm endocarps evidence are indicated. We assumed that charred oil palm endocarps found in combination with potsherds were consumed during the occupation of the sites (Morin-Rivat et al., 2016).

| **Country** | **Name** | **Fig. 1** | **Latitude** | **Longitude** | **Dated mat.** | **Identified sp.** | **Lab nr** | **BP** | **±** | **Cal. BP** | **Cal. AD** | **Reference** | **Pottery** | **Smelting** | **Salt** | **Oil palm** |
| --- | --- | --- | --- | --- | --- | --- | --- | --- | --- | --- | --- | --- | --- | --- | --- | --- |
| C | Bagofit | 1 | 4.00000 | 13.12000 | charcoal | . | Erl-12252 | 268 | 39 | 463-0 | 1719 | Meyer et al., 2009 | x | x | . | . |
| C | Mindourou 2 | 2 | 3.64660 | 13.52290 | seed | *E. g.* | KIA-45496 | 1050 | 25 | 1049-925 | 875 | Morin-Rivat et al., 2014 | . | . | . | x |
| C | Mindourou 3 | 3 | 3.09592 | 13.96362 | seed | . | Poz-49314 | 205 | 30 | 305-0 | 1798 | Morin-Rivat et al., 2016 | . | . | . | . |
| C | Mindourou 4 | 4 | 3.20084 | 13.95598 | charcoal | . | Poz-49316 | 210 | 30 | 305-0 | 1798 | Morin-Rivat et al., 2016 | . | . | . | . |
| C | Mbang 1 | 5 | 4.02815 | 14.17808 | seed | *E. g.* | Poz-62627 | 860 | 30 | 901-695 | 1152 | Morin-Rivat et al., 2016 | . | . | . | x |
| C | Mbang 2 | 6 | 4.00818 | 14.18169 | charcoal | . | Poz-62629 | 175 | 35 | 298-0 | 1801 | Morin-Rivat et al., 2016 | . | . | . | . |
| C | Mbang 3 | 7 | 3.87331 | 14.21665 | charcoal | . | Poz-62630 | 305 | 30 | 462-300 | 1570 | Morin-Rivat et al., 2016 | . | . | . | . |
| C | Mbang 4 | 8 | 3.84654 | 14.22167 | seed | *E. g.* | Poz-62634 | 360 | 30 | 500-315 | 1542 | Morin-Rivat et al., 2016 | . | . | . | x |
| C | Bali River 1 | 9 | 3.73690 | 14.73840 | charcoal | . | Poz-41774 | 110 | 25 | 268-15 | 1807 | Morin-Rivat et al., 2014 | . | . | . | . |
| C | Bali River 2 | 10 | 3.76250 | 14.79306 | charcoal | . | Poz-41775 | 590 | 30 | 652-537 | 1356 | Morin-Rivat et al., 2014 | x | . | . | . |
| C | Mbang 5 | 11 | 3.45867 | 14.31220 | charcoal | . | Poz-62638 | 260 | 30 | 431-0 | 1735 | Morin-Rivat et al., 2016 | . | . | . | . |
| C | Mbang 6 | 11 | 3.46071 | 14.31181 | charcoal | . | Poz-62637 | 140 | 30 | 281-6 | 1807 | Morin-Rivat et al., 2016 | . | . | . | . |
| C | Mbang 7 | 11 | 3.45206 | 14.31393 | charcoal | . | Poz-62640 | 80 | 30 | 260-25 | 1808 | Morin-Rivat et al., 2016 | . | . | . | . |
| C | Messok 3 | 12 | 3.07050 | 14.35570 | charcoal | . | KIA-38942 | 195 | 30 | 302-0 | 1799 | Morin-Rivat et al., 2014 | . | . | . | . |
| C | Messok 3 | 12 | 3.07052 | 14.35566 | charcoal | . | KIA-38934 | 205 | 30 | 305-0 | 1798 | Morin-Rivat et al., 2014 | x | . | . | . |
| C | Ndangayé Bai | 13 | 2.38333 | 15.80000 | . | . | KIA-31773 | 1045 | 30 | 1050-921 | 963 | Oslisly et al., 2013b | x | . | x | . |
| C | Djembé Fosse 1 | 14 | 2.18333 | 16.06667 | . | . | Gif-12476 | 585 | 35 | 652-534 | 1357 | Oslisly et al., 2013b | . | . | . | . |
| C | Bolo Bai 1B | 15 | 2.16667 | 15.71667 | . | . | Poz-45694 | 1160 | 30 | 1177-983 | 870 | Oslisly et al., 2013b | x | . | x | . |
| C | Bolo Bai 1C | 16 | 2.15000 | 15.71667 | . | . | KIA-31772 | 948 | 20 | 925-796 | 1090 | Oslisly et al., 2013b | x | . | x | . |
| C | Ngoko Island | 17 | 1.93333 | 15.58333 | . | . | Poz-45692 | 145 | 30 | 283-2 | 1808 | Oslisly et al., 2013b | . | . | . | . |
| C | Mokounounou 1a | 18 | 1.93333 | 15.33333 | . | . | KI-4612 | 470 | 65 | 641-318 | 1471 | Oslisly et al., 2013b | x | . | x | . |
| C | Mokounounou 1b | 18 | 1.93333 | 15.33333 | . | . | KI-4613 | 410 | 55 | 531-315 | 1527 | Oslisly et al., 2013b | x | . | x | . |
| RC | Upper Esimbi | 19 | 2.27600 | 16.42700 | seed | *E. g.* | Beta-75799 | 1150 | 70 | 1255-933 | 856 | Fay, 1997 | . | . | . | x |
| RC | Mid Esimbi | 20 | 2.34100 | 16.34400 | seed | *E. g.* | Beta-75800 | 990 | 80 | 1064-730 | 1053 | Fay, 1997 | . | . | . | x |
| RC | Mid Esimbi | 20 | 2.34100 | 16.34400 | seed | *E. g.* | Beta-75802 | 1030 | 80 | 1174-767 | 980 | Fay, 1997 | . | . | . | x |
| RC | Mid Esimbi | 20 | 2.34100 | 16.34400 | seed | *E. g.* | Beta-75805 | 1110 | 70 | 1237-913 | 965 | Fay, 1997 | . | . | . | x |
| RC | Bomassa 1 | 21 | 2.16300 | 16.19490 | charcoal | . | KIA-37683 | 200 | 25 | 299-0 | 1800 | Morin-Rivat et al., 2014 | . | . | . | . |
| RC | Loundoungou | 22 | 2.43073 | 16.97062 | seed | *E. g.* | Poz-41787 | 90 | 30 | 266-22 | 1806 | Morin-Rivat et al., 2014 | . | . | . | x |
| CAR | Ndakan | 23 | 2.21000 | 16.90000 | pottery | . | X367 (OSL) | 1.39 | 0.99 | 920/460 | . | Brnčić, 2003 | x | . | . | . |
| CAR | Ndakan | 23 | 2.21000 | 16.90000 | pottery | . | X368 (OSL) | 1.15 | 0.27 | 380/760 | . | Brnčić, 2003 | x | . | . | . |
| RC | Mokobo | 24 | 2.01560 | 16.65310 | seed | *N.* sp. | KIA-37685 | 215 | 25 | 305-0 | 1797 | Morin-Rivat et al., 2014 | . | . | . | . |
| RC | Djaka River | 25 | 1.71851 | 16.46576 | charcoal | . | Poz-38696 | 335 | 35 | 482-308 | 1556 | Morin-Rivat et al., 2014 | x | . | . | . |
| RC | Komo River | 26 | 1.68030 | 16.84610 | seed | *E. g.* | Poz-41780 | 1200 | 30 | 1236-1010 | 827 | Morin-Rivat et al., 2014 | . | . | . | x |
| RC | Ngombé | 27 | 1.43532 | 16.17317 | charcoal | . | Poz-38703 | 675 | 30 | 679-560 | 1331 | Morin-Rivat et al., 2014 | x | x | . | . |
| RC | Pikounda 2 | 28 | 1.29318 | 16.20120 | seed | *E. g.* | Poz-41772 | 520 | 30 | 626-507 | 1383 | Morin-Rivat et al., 2014 | x | . | . | x |
| RC | Pokola 2 | 29 | 1.24669 | 16.66761 | seed | *E. g.* | KIA-34142 | 620 | 25 | 658-5551 | 1346 | Morin-Rivat et al., 2014 | . | . | . | x |
| RC | Landjoué | 30 | 0.76502 | 15.36163 | seed | *E. g.* | Poz-41781 | 290 | 30 | 458-288 | 1577 | Morin-Rivat et al., 2014 | x | . | . | x |
| CAR | Sabele 1 | 31 | 3.33333 | 16.16667 | charcoal | . | Ly-5921 | 630 | 45 | 667-546 | 1343 | Lanfranchi et al., 1998 | . | x | . | . |
| CAR | Sabele 2 | 31 | 3.33333 | 16.16667 | charcoal | . | Ly-5922 | 715 | 35 | 726-564 | 1305 | Lanfranchi et al., 1998 | . | x | . | . |
| RC | Lopola | 32 | 2.99360 | 17.30260 | charcoal | . | Poz-41784 | 140 | 25 | 281-6 | 1806 | Morin-Rivat et al., 2014 | . | . | . | . |
| RC | Ebaleki River | 33 | 3.21733 | 17.51133 | charcoal | . | Poz-38697 | 315 | 30 | 466-302 | 1566 | Morin-Rivat et al., 2014 | x | . | . | x |
| CAR | Bagbaya BB01 | 34 | 3.50000 | 17.28000 | charcoal | . | AA94530 | 168 | 35 | 291-0 | 1804 | Lupo et al., 2015 | . | x | . | . |
| CAR | Bagbaya BB01 | 34 | 3.50000 | 17.28000 | charcoal | . | AA94531 | 207 | 35 | 309-0 | 1795 | Lupo et al., 2015 | . | x | . | . |
| CAR | Bagbaya BB01 | 34 | 3.50000 | 17.28000 | charcoal | . | AA94529 | 215 | 34 | 310-0 | 1795 | Lupo et al., 2015 | . | x | . | . |
| CAR | Bagbaya BB03 | 34 | 3.50000 | 17.28000 | charcoal | . | AA94532 | 148 | 34 | 284-0 | 1808 | Lupo et al., 2015 | . | x | . | . |
| CAR | Bagbaya BB05 | 34 | 3.50000 | 17.28000 | charcoal | . | AA94534 | 187 | 34 | 303-0 | 1799 | Lupo et al., 2015 | . | x | . | . |
| CAR | Bagbaya BB05 | 34 | 3.50000 | 17.28000 | charcoal | . | AA94533 | 231 | 34 | 422-0 | 1739 | Lupo et al., 2015 | . | x | . | . |
| CAR | Bagbaya ND01 | 34 | 3.50000 | 17.28000 | charcoal | . | AA95437 | 160 | 35 | 287-0 | 1807 | Lupo et al., 2015 | . | x | . | . |
| CAR | Bagbaya ND02 | 34 | 3.50000 | 17.28000 | charcoal | . | AA94538 | 242 | 34 | 429-0 | 1736 | Lupo et al., 2015 | . | x | . | . |
| CAR | Bagbaya NG01 | 34 | 3.50000 | 17.28000 | charcoal | . | AA94539 | 217 | 48 | 429-0 | 1735 | Lupo et al., 2015 | . | x | . | . |
| CAR | Bagbaya NZ03 | 34 | 3.50000 | 17.28000 | charcoal | . | AA94541 | 494 | 34 | 621-497 | 1391 | Lupo et al., 2015 | . | x | . | . |
| CAR | Bagbaya NZ03 | 34 | 3.50000 | 17.28000 | charcoal | . | AA94542 | 593 | 34 | 654-538 | 1355 | Lupo et al., 2015 | . | x | . | . |
| CAR | Bagbaya NZ03 | 34 | 3.50000 | 17.28000 | charcoal | . | AA94540 | 706 | 35 | 701-561 | 1319 | Lupo et al., 2015 | . | x | . | . |
| CAR | Bagbaya OB01 | 34 | 3.50000 | 17.28000 | charcoal | . | AA94543 | 152 | 35 | 285-0 | 1808 | Lupo et al., 2015 | . | x | . | . |
| CAR | Bagbaya OB02 | 34 | 3.50000 | 17.28000 | charcoal | . | AA94544 | 210 | 34 | 309-0 | 1795 | Lupo et al., 2015 | . | x | . | . |
| CAR | Bagbaya OB05 | 34 | 3.50000 | 17.28000 | charcoal | . | AA94546 | 131 | 34 | 280-7 | 1806 | Lupo et al., 2015 | . | x | . | . |
| CAR | Bagbaya OB05 | 34 | 3.50000 | 17.28000 | charcoal | . | AA94545 | 188 | 39 | 305-0 | 1797 | Lupo et al., 2015 | . | x | . | . |
| CAR | Bagbaya OB06 | 34 | 3.50000 | 17.28000 | charcoal | . | AA94547 | 131 | 34 | 280-7 | 1806 | Lupo et al., 2015 | . | x | . | . |
| CAR | Ngara | 35 | 4.03000 | 18.38000 | . | . | Ly-5919 | 330 | 40 | 485-305 | 1555 | Moga, 2008 | . | x | . | . |
| CAR | Lingbangbo | 36 | 3.81667 | 18.53333 | charcoal | . | OBDY-463 | 70 | 120 | 424-0 | 1738 | Moga, 2008 | . | x | . | . |
| CAR | Lingbangbo | 36 | 3.81667 | 18.53333 | charcoal | . | OBDY-464 | 110 | 80 | 285-0 | 1808 | Moga, 2008 | . | x | . | . |
| CAR | Lingbangbo | 36 | 3.81667 | 18.53333 | charcoal | . | OBDY-255 | 430 | 180 | 689-0 | 1605 | Moga, 2008 | . | x | . | . |
| CAR | Lingbangbo | 36 | 3.81667 | 18.53333 | charcoal | . | OBDY-582 | 559 | 77 | 673-493 | 1367 | Moga, 2008 | . | x | . | . |
| CAR | Sikilongo | 37 | 3.65000 | 18.56000 | charcoal | . | OBDY-303 | 870 | 210 | 1256-518 | 1063 | Moga, 2008 | . | x | . | . |
| CAR | Mondongué | 38 | 3.65000 | 18.56000 | charcoal | . | OBDY-253 | 140 | 100 | 428-0 | 1736 | Moga, 2008 | . | x | . | . |
