# Supplementary material for: Present-day central African forest is a legacy of the 19th century human history
Source: eLife. 2017 Jan 17;6:e20343. doi: 10.7554/eLife.20343 (PMC5241113; doi:10.7554/eLife.20343)
Supplement: Supplementary file 7. — DOI: http://dx.doi.org/10.7554/eLife.20343.014 [file elife-20343-supp7.docx]

**Supplementary file 7**

**SQL codes for the Bayesian analysis of the radiocarbon dates.**

Plot()

 {

Sequence()
{
  Sigma_Boundary(“Start”);

 Sum(“Phase”)

 {

R_Date(BP(calBP(70)), 120) +N(0,10);

R_Date(BP(calBP(80)), 30) +N(0,10);

R_Date(BP(calBP(90)), 30) +N(0,10);

R_Date(BP(calBP(110)), 25) +N(0,10);

R_Date(BP(calBP(110)), 80) +N(0,10);

R_Date(BP(calBP(131)), 34) +N(0,10);

R_Date(BP(calBP(131)), 34) +N(0,10);

R_Date(BP(calBP(140)), 30) +N(0,10);

R_Date(BP(calBP(140)), 25) +N(0,10);

R_Date(BP(calBP(140)), 100) +N(0,10);

R_Date(BP(calBP(145)), 30) +N(0,10);

R_Date(BP(calBP(148)), 34) +N(0,10);

R_Date(BP(calBP(152)), 35) +N(0,10);

R_Date(BP(calBP(160)), 35) +N(0,10);

R_Date(BP(calBP(168)), 35) +N(0,10);

R_Date(BP(calBP(175)), 35) +N(0,10);

R_Date(BP(calBP(187)), 34) +N(0,10);

R_Date(BP(calBP(188)), 39) +N(0,10);

R_Date(BP(calBP(195)), 30) +N(0,10);

R_Date(BP(calBP(200)), 25) +N(0,10);

R_Date(BP(calBP(205)), 30) +N(0,10);

R_Date(BP(calBP(205)), 30) +N(0,10);

R_Date(BP(calBP(207)), 35) +N(0,10);

R_Date(BP(calBP(210)), 30) +N(0,10);

R_Date(BP(calBP(210)), 34) +N(0,10);

R_Date(BP(calBP(215)), 25) +N(0,10);

R_Date(BP(calBP(215)), 34) +N(0,10);

R_Date(BP(calBP(217)), 48) +N(0,10);

R_Date(BP(calBP(231)), 34) +N(0,10);

R_Date(BP(calBP(242)), 34) +N(0,10);

R_Date(BP(calBP(260)), 30) +N(0,10);

R_Date(BP(calBP(268)), 39) +N(0,10);

R_Date(BP(calBP(290)), 30) +N(0,10);

R_Date(BP(calBP(305)), 30) +N(0,10);

R_Date(BP(calBP(315)), 30) +N(0,10);

R_Date(BP(calBP(330)), 40) +N(0,10);

R_Date(BP(calBP(335)), 35) +N(0,10);

R_Date(BP(calBP(360)), 30) +N(0,10);

R_Date(BP(calBP(410)), 55) +N(0,10);

R_Date(BP(calBP(430)), 180) +N(0,10);

R_Date(BP(calBP(470)), 65) +N(0,10);

R_Date(BP(calBP(494)), 34) +N(0,10);

R_Date(BP(calBP(520)), 30) +N(0,10);

R_Date(BP(calBP(559)), 77) +N(0,10);

R_Date(BP(calBP(585)), 35) +N(0,10);

R_Date(BP(calBP(590)), 30) +N(0,10);

R_Date(BP(calBP(593)), 34) +N(0,10);

R_Date(BP(calBP(620)), 25) +N(0,10);

R_Date(BP(calBP(630)), 45) +N(0,10);

R_Date(BP(calBP(675)), 30) +N(0,10);

R_Date(BP(calBP(706)), 35) +N(0,10);

R_Date(BP(calBP(715)), 35) +N(0,10);

R_Date(BP(calBP(860)), 30) +N(0,10);

R_Date(BP(calBP(870)), 210) +N(0,10);

R_Date(BP(calBP(948)), 20) +N(0,10);

R_Date(BP(calBP(990)), 80) +N(0,10);

R_Date(BP(calBP(1030)), 80) +N(0,10);

R_Date(BP(calBP(1045)), 30) +N(0,10);

R_Date(BP(calBP(1050)), 25) +N(0,10);

R_Date(BP(calBP(1110)), 70) +N(0,10);

R_Date(BP(calBP(1150)), 70) +N(0,10);

R_Date(BP(calBP(1160)), 30) +N(0,10);

R_Date(BP(calBP(1200)), 30) +N(0,10);

}; 
  Sigma_Boundary(“End”); 
 };

};
