# Supplementary material for: Present-day central African forest is a legacy of the 19th century human history
Source: eLife. 2017 Jan 17;6:e20343. doi: 10.7554/eLife.20343 (PMC5241113; doi:10.7554/eLife.20343)
Supplement: Supplementary file 8. — Precise dates or time spans are related to local or more general events. References are indicated. DOI: http://dx.doi.org/10.7554/eLife.20343.015 [file elife-20343-supp8.docx]

**Supplementary file 8**

**Chronology of the historical events from the beginning of the 15^th^ century to the present occurring or influencing human populations in the SRI.**

Precise dates or time spans are related to local or more general events. References are indicated.

| **Date** | **Event** | **References** |
| --- | --- | --- |
| **1400-1650** | Kongo Kingdom in the current Rep. of the Congo and Angola. Slave trade by the Portuguese caused the kingdom to collapse by depopulating and destabilizing the area | Gendreau, 2010; Stock, 2013 |
| **1441-1870** | Slave trade, including Triangular trade, with primary period of slaves transported across the Atlantic between 1700 and 1870 (80%) | Stock, 2013 |
| **1800-1850** | In central Africa, slaves were captured far inland (30,000 slaves sent per year) | Manning and Akyeampong, 2006 |
| **~ 1840-1900** | Southward movements of populations into the forests to escape the Fulbe's (and affiliates') slave-raiding: social disorganization and overpopulation in the Sangha region, particularly along rivers | Copet-Rougier, 1998; Robineau, 1967; Giles-Vernick, 2000 |
|  | Interethnic wars then social structuration at the end of the 19th c. through marriage exchanges (alliances) in the SRI | Copet-Rougier, 1998 |
|  | Economic attraction for the Sangha region: fertile soils, natural resources, e.g., iron, and thriving trade | Copet-Rougier, 1998 |
| **1875** | Beginning of the exploration of southeastern Cameroon and of the Congo | Burnham, 1996 |
| **~ 1880** | Beginning of the "Scramble for Africa" | Stock, 2013 |
| **1884-1885** | Berlin Conference (Partition of Africa): on paper delimitation of the French/German border | Robineau, 1967 |
| **1885-1890** | Arrival of the first Europeans in central Africa (French and Germans) | Robineau, 1967 |
| **1887** | German military campaign near Abong-Mbang: seething of the Dja populations | Robineau, 1967 |
| **1891** | First arrival of French explorers Gaillard and Fourneau in the Upper-Sangha region. Creation of the Ouesso station | Copet-Rougier, 1998; Robineau, 1967 |
| **1892** | De Brazza is appointed as Commissioner General of the Congo | Burnham, 1996 |
|  | De Brazza describes the Upper-Sangha region as densely populated and rich in resources with a well-organized trade between agriculturists and pastoralists | Copet-Rougier, 1998; Coquery-Vidrovitch, 1998 |
|  | Permanent French occupation of the region | Copet-Rougier, 1998 |
| **1894** | Franco-German Treaty delimiting the border | Burnham, 1996; Robineau, 1967 |
|  | Establishment of the guarding posts from Koundé (north) to Nola (south) | Burnham, 1996 |
| **1894-1896** | Insurrection then repression of the local populations and the Fulbe slave-raiders by the colonial power, particularly near Nola | Copet-Rougier, 1998; Coquery-Vidrovitch, 1998 |
| **1897** | Reduction of the French colonial occupation to the post of Carnot only | Copet-Rougier, 1998 |
| **1898-1899** | Twelve French companies dispute the Upper Congo: 30-year concessions to exploit the resources and develop communications. Creation of factories | Vennetier, 1963; Coquery-Vidrovitch, 1998 |
| **1898** | Arrival of the Germans at Ouesso (von Carnapp, Plehn, and von Stein) followed by expansion to the north (Yokadouma and Bertoua) | Copet-Rougier, 1998 |
| **1899** | Foundation of the Sangha-Ngoko station at Mouloundou by the Germans. Beginning of the tensions between the Germans and the French | Copet-Rougier, 1998 |
| **~ 1900** | Gathering of the populations in the European trading centers (admin. or military posts) and in traditional centers (implantation of colonial factories and trading posts) | Robineau, 1967 |
| **1902-1909** | Insurrection of the local populations against the companies, followed by repression | Coquery-Vidrovitch, 1998 |
| **1905-1906** | Commercial conflicts between French and German companies in the absence of a clear border | Robineau, 1967 |
| **1905-1907** | Denunciation of brutalities and murders of locals by the concessionaires | Coquery-Vidrovitch, 1998 |
| **1907-1911** | Arming of the populations by the French and German colonists, interethnic conflicts and looting of the factories, military operations with destruction of villages | Robineau, 1967 |
| **Before 1910** | People formerly settled along the rivers: Sangha, Dja, Djouah, and Aïna; empty zones in the forests btw. the Dja and Boumba rivers | Robineau, 1967 |
| **1910-1930** | Forced population displacements, emptying of the riverbanks | Robineau, 1967 |
| **1911** | New physical delimitation of the Franco-German border more southward in exchange for possessions in Morocco | Robineau, 1967 |
| **1911-1914** | France cedes the Sangha Valley to the Germans. French possessions are spilt apart from the Sangha River | Coquery-Vidrovitch, 1998 |
| **1913** | Evacuation of the French posts and factories: draining of the populations to Ouesso | Robineau, 1967 |
| **1914-1918** | Involvement of local populations in WWI (armed), French/German clashes over control of the region, the populations flee | Robineau, 1967, Giles-Vernick, 2000 |
| **1919** | Paris Peace Conference: recovery by France of the German possessions ceded in 1911, with French Equatorial Africa (AEF) included | Kaspi, 1971 |
| **1920s** | Implantation of extensive oil palm and coffee plantations in the Sangha River Interval | Giles-Vernick, 2000 |
| **1920-1930** | Permanent settlement of the colonial posts, layout of new trails and roads with forced labor, more accurate census of the populations, increased role of the local chiefs | Robineau, 1967, Freed, 2010 |
|  | Displacements of villages along the main road axes only (*cantonment* in French), destruction of the ancient villages, collapse of the Bakwélé group | Robineau, 1967 |
| **1921-1932** | Enrollment for the construction of the railway (Congo-Ocean): conflicts btw. rubber companies and the colonial admin (AEF; drainage of the workers) | Robineau, 1967 |
|  | Desertion of the villages, flight of people to Cameroon (particularly the Djem led by their chief Angoula) | Robineau, 1967 |
|  | Mortality of workers in the construction of the railway: up to 30.3 % (accidents and epidemics) | Robineau, 1967 |
| **~1923-1925** | Christian missions settled in the Sangha River Interval | Giles-Vernick, 2000 |
| **1923-1945** | Exploitation of the natural rubber (in 10-y concessions), only profitable during WWII (war effort); new trails to export the rubber production | Robineau, 1967 |
| **1925-1928** | Decline in the population from 20,000 to 15,000 btw. two censuses | Robineau, 1967 |
| **1928-1930** | Great insurrection in the Upper-Sangha, including Cameroon and northern Congo | Coquery-Vidrovitch, 1998 |
| **~1930** | Beginning of the rural exodus | Vennetier, 1963 |
| **1938** | 22% of the population suffers from sleeping sickness in the Nola subdivision | Robineau, 1967 |
| **1939-1945** | Increasing rubber exploitation during World War II | Coquery-Vidrovitch, 1998 |
| **1945-1957** | Collapse of the rubber production and gold extraction, and failure of the coffee plantations: development of the oil palm (e.g., in Ouesso) and cocoa plantations, timber exploitation | Robineau, 1967, Coquery-Vidrovitch, 1998 |
|  | Beginning the abandonment of road construction projects | Robineau, 1967, Coquery-Vidrovitch, 1998 |
| **1960** | Independence for three of the studied countries | Robineau, 1967 |
| **1964** | Massive immigration of workers to Ouesso | Robineau, 1967 |
| **1970-1976** | Modern timber exploitation in central Africa | Vennetier, 1963; Laporte et al., 2007 |
| **1980s-1990s** | Social impacts of economic and environmental interventions (reserves and parks). Employment of workers in timber companies (concessions). End of agricultural activities in the forest | Giles-Vernick, 2000 |
